# Determination of the Effects of a Series of Ten Whole-Body Cryostimulation Sessions on Physiological Responses to Exercise and Skin Temperature Behavior following Exercise in Elite Athletes

**DOI:** 10.3390/jcm12196159

**Published:** 2023-09-24

**Authors:** Ilona Pokora, Zofia Drzazga, Piotr Wyderka, Mariusz Binek

**Affiliations:** 1 Department of Physiology, Institute of Sport Sciences, The Jerzy Kukuczka Academy of Physical Education in Katowice, Mikołowska 72a, 40-065 Katowice, Poland; 2 The Silesian Centre for Education and Interdisciplinary Research, Faculty of Science and Technology, University of Silesia in Katowice, 75 Pułku Piechoty 1A, 41-500 Chorzow, Poland

**Keywords:** whole-body cryostimulation, exercise, athletes, skin temperature

## Abstract

The present study investigated the effects of a series of 10 whole-body cryostimulation (WBC) sessions (3 min; −110 °C) on physiological and thermal responses to a submaximal exercise test in 17 elite athletes. Participants performed an exercise test twice at similar levels of intensity before and after a series of ten WBC sessions. Before and during the test, each participant’s oxygen uptake (VO_2_), heart rate (HR), internal temperature (Ti), and skin temperature in selected areas of the skin were measured, and the mean arterial pressure (MAP), physiological strain index (PSI), and mean skin temperature (Tsk) were calculated. The results show that during exercise, increases in Ti and the PSI were significantly lower after the WBC sessions, and although there were no significant changes in HR or the MAP, the Tsk was significantly higher. Following exercise, an increase in skin temperature asymmetry over the lower-body muscles was detected. A series of WBC sessions induced a tendency toward a decrease in temperature asymmetry over the thigh muscles. In conclusion, a series of ten WBC sessions does not induce significant modifications in physiological variables but does influence the PSI and Ti during exercise. Moreover, a series of ten WBC sessions influences the distribution of skin temperature and the magnitude of temperature asymmetries in the early phase of recovery.

## 1. Introduction

Whole-body cryostimulation (WBC) is defined as extreme cold therapy or cold stimulation involving the brief (3–4 min) exposure of large areas of the body to dry, very cold (−120 °C) air [1,2,3]. Whole-body cryostimulation (WBC) does not decrease the internal body temperature but only reduces the skin temperature, and it does not lead to hypothermia [4]. Cryostimulation is utilized as a physical intervention in the treatment of injury and exercise recovery, and it is tolerated well by athletes [5,6]. In a sports context, WBC is applied in the form of repetitive whole-body cryostimulation (WBC) during training programs to accelerate post-exercise recovery [1,7,8,9,10,11,12,13], reduce metabolism, improve exercise performance [11,14], reduce the perception of pain and fatigue [1,12], attenuate exercise-induced muscle damage and inflammation [8,13], enhance the regenerative response [15,16] and improve the magnitude of performance supercompensation [17].

Elite-level athletic participation necessitates recovery from many physiological stressors, including fatigue to the musculoskeletal, nervous and metabolic systems [18]. Athletic participation may also result in exercise-induced muscle damage (EIMD), which may lead to delayed-onset muscle soreness (DOMS) and decrements in subsequent performance [19]. Different studies have demonstrated the effectiveness of whole-body cryostimulation (WBC) in supporting recovery processes. In a review written by Rose et al. [11], it was shown that the application of WBC following intensive exercise had an analgesic effect and improved physical performance. In the context of recovery after physical exercise, WBC exposure and the resulting decrease in muscle and skin temperature may decrease muscle enzyme activities, metabolism, inflammation, and secondary muscle damage after exercise (delayed onset muscle soreness (DOMS)), which can promote recovery [5,20,21]. This form of temperature treatment can be used at any stage of the training process, including during the preparatory phase [22], during the recovery period [13], during simulated 7-day tapering [17] and after the competitive season [23,24], especially in scenarios in which the implementation of recovery strategies can accelerate a return to optimal performance [6].

Cross-country skiing is a very demanding endurance sport. The competitive period is particularly taxing on the body, after which athletes have a very short recovery period. The specific training and recovery processes undertaken by cross-country skiers can be challenging for coaches and physiotherapists as they must determine optimal training programs for their athletes during specific periods and, at the same time, secure appropriate regeneration methods. To date, there is insufficient knowledge of recovery methods that are suitable for elite athletes, especially with respect to supporting recovery practices within a professional training program. It appears that for elite athletes, the inclusion of WBC series during the transition period of a training program (when the training volume is low) can be an effective way to enhance and accelerate recovery and improve the elimination of fatigue after the competitive season. Although WBC has become very popular with both recreational and elite athletes, to date there is a limited body of evidence regarding its efficacy, and empirical data detailing the potential mechanism(s) through which this treatment could be effective are sparse.

Physiological responses to cold exposure depend on the length of exposure, the medium of cold exposure (air versus water), anthropometric body characteristics and the number of previous exposures. An immediate and generalized decrease in skin temperature (Tsk) is the initial effect of cryogenic exposure [25,26]. This reaction is governed by the sympathetic nervous system, leading to superficial veno- and vasoconstriction. These vascular changes result in the translocation of blood from cutaneous vessels to deeper veins and from the periphery to the core, as well as in increases in the mean arterial blood pressure, cardiac output and stroke volume, with a consequent decrease in heart rate [27,28]. During the second phase, following the procedure, vasodilation and an increase in the perfusion of skin take place.

Physiological adjustments to repeated cold exposure are either insulative (a reduction in the mean skin temperature) or metabolic (shivering or non-shivering thermogenesis). Young [29] postulated that the type of cold adaptation response is dependent on the degree of cooling of the body. The prevailing view is that if repeated brief exposures to cold are not associated with a significant reduction in internal body temperature, they may induce a specific form of cold adaptation classified as cold habituation [30,31,32]. Cold habituation is a form of cold adaptation that involves a reduced response to or the perception of repeated cold stimulation [33] and can develop after just a few repeated brief exposures to cold air or water. The typical features of habituation to cold include habituation to shivering (e.g., a delayed onset and reduced metabolism), higher skin temperature, the suppression of vasoconstriction, a less intense cold sensation, lower blood pressure and the release of norepinephrine in response to cold exposure, as well as increased parasympathetic activation [27,32,34].

Physical exertion causes an intensity-dependent decrease in the activity of the parasympathetic nervous system and an increase in the activity of the sympathetic nervous system, so it is desirable to quickly restore parasympathetic activity after exercise, with which habituation to cold can help [27,34]. Although the physiological effects of WBC mostly show positive results and are already supported by a large body of evidence, there is currently little information on changes in the body’s response to exercise after a series of ten cryostimulation sessions.

Given the aforementioned characteristics of cold-induced habituation, it seemed reasonable to investigate whether a series of 10 WBC sessions would affect the physiological characteristics of the body’s response to submaximal exercise in male cross-country skiers, and whether it would be accompanied by changes in the post-exercise skin temperature.

During exercise, the production of metabolic heat and the circulatory and thermoregulatory system loads increase [35]. Since the hemodynamic and thermoregulatory adjustments triggered during exercise to maintain thermal equilibrium can modify the Tsk, and considering that the Tsk plays the fundamental role of regulating heat exchange via convection, radiation and evaporation [36], measuring these alterations in the Tsk in response to exercise may provide valuable information on the effectiveness of skin thermoregulation [37]. An important tool that provides data on the heat radiated by the body is infrared thermography (IRT). The production of heat that occurs in a working muscle is transferred to the superficial tissue and can be measured as the skin surface temperature (Tsk) via infrared thermography (IRT). Therefore, IRT can play an important role as a noninvasive and non-contact method of diagnosis in sports science [38,39] and has become a popular technique for determining the temperature of human skin at rest and during exercise [36,37,40,41,42,43]. There is limited evidence that habituation to cold can alter skin temperature responses to exercise. An examination of this issue may lead to new discoveries about the adaptations and physiological mechanisms that can be supported following cold therapy. Although IRT techniques are used in physical activity and sports research and IRT devices are some of the most common devices for monitoring the effects of cryotherapy [44,45,46], there is a lack of knowledge and data about how the Tsk behaves immediately after exercise in elite athletes after a series of ten cryostimulation sessions.

Considering the effects of a series of ten WBC sessions on physiological responses to exercise in humans, we hypothesized that a series of ten cryostimulation sessions interspersed across typical training in the transition phase of an annual training program can induce some cold habituation adaptations, reduce physiological strain during exercise and optimize post-exercise recovery. Therefore, the objectives of this study were twofold: (a) to examine the effect of repeated WBC sessions on indexes such as stress hormone concentrations, internal and skin temperature and the heart rate, both at rest and in response to exercise; and (b) to investigate whether repeated WBC treatments can affect skin temperature behavior following exercise in elite cross-country skiers.

## 2. Materials and Methods

### 2.1. Ethical Approval

This research was conducted in accordance with the Declaration of Helsinki, and the research methodology was approved by the Bioethical Committee of the Regional Medical Chamber in Katowice (U2/KB/2016). The participants were informed in detail about the purpose and course of the study and about the possibility to withdraw from participation in the project at any stage without giving justification. All of the subjects read the written information about the course of the research and the clothing and behavior required during the WBC procedures. The participants provided their written consent to voluntarily participate in the experiment.

### 2.2. Participants

Seventeen elite athletes (male cross-country skiers) participated in this study. All subjects were members of the Polish National Ski Team. The inclusion criteria required that all participants had a professional training history of at least ten years. The participants mentioned in this study were elite athletes (cross-country skiers), and prior to the study, they all provided written consent to their inclusion in the study, and each submitted an Athlete Biological Passport (ABP) for review.

The exclusion criteria for this study included the following absolute contraindications to cryostimulation: hypertension, cold intolerance and Raynaud’s disease.

The subjects’ characteristics are presented in Table 1.

### 2.3. Experimental Design

The experimental procedures were previously used in our study [47], with subtle differences noted below and with cross-references provided for additional details (Table 2).

#### 2.3.1. Preliminary Study

Before the first trial, all subjects completed a familiarization session, a graded exercise test (GXT) to volitional fatigue (VO_2_max) and an anthropometric assessment.

The following somatic measurements were obtained: the height was measured using a stadiometer (Seca 217, Hamburg, Germany), and the body mass (BM) and body composition (BMI—body mass index; FM—total body fat; FFM—fat-free mass) were assessed using a bioimpedance analysis (Inbody 220, Korea).

The determinant of aerobic capacity is VO_2_max. It was determined by a graded exercise test (GXT). For baseline testing, the participants attended a laboratory in the Research Center for Sports, Academy of Physical Education, Katowice, to complete a graded exercise test (GXT). The maximal oxygen uptake (VO_2_max) was measured by connecting the participants to a breath-by-breath gas analyzer (MetaLyzer 3B-R2, Leipzig, Germany) and having them perform an incremental exercise test on a treadmill (Cosmed, Germany). The treadmill speed was increased by 2 km/h every 3 min until a running speed of 14 km/h was reached, after which the treadmill inclination was raised in 2.5-degree increments every 3 min, and the test continued until voluntary exhaustion, as described previously [47,48]. During the GXT test, the heart rate (HR), oxygen uptake (VO_2_) and blood lactate concentration were recorded. Based on the blood lactate concentration, the anaerobic threshold was determined via the D-max method [49]. The HR corresponding to the anaerobic threshold (LT) exercise intensity was determined (HR-LT). The HR-LT was used to appropriate the estimation of exercise intensity, which was used during the experimental tests. Approximately 7 days after the preliminary exercise test, the participants completed the main exercise test at the predetermined intensity, below the HR-LT.

#### 2.3.2. Experimental Exercise Test and Whole-Body Cryostimulation

The study was conducted from April to June during the transition phase (TP) of an annual training program (during the recovery mesocycle). During the annual training cycle, the athletes (cross-country skiers) have a very short recovery period for full regeneration. At this stage of the training program, the training volume is low and averages 60–80 min a day. During the study period, the participants recorded the duration and intensity of their training prior to the experiment and were instructed to attempt to repeat this training throughout the study.

This exercise study was conducted at the Research Center for Sports, Academy of Physical Education, Katowice, before and after a series of ten cryostimulation sessions.

Submaximal Exercise (SM Test):

The main physical effort used in this study was one hour of uninterrupted running exercise (SM) performed in thermoneutral conditions (an ambient temperature of 21–24 °C and a relative humidity of 45–55%). The participants performed the continuous SM test twice: before (A) and after a series of 10 cryostimulation (B) sessions. The SM test was performed at the predetermined intensity, below the HR-LT, for up to 60 min (Table 1). Two similar exercise tests at a given intensity (% of VO_2_max) allowed for the determination of the subjects’ response to similar physical efforts before and after WBC. No fluid was consumed during the exercise test.

The Whole-Body Cryostimulation Procedure:

The participants were subjected to 10 WBC treatments in the cryogenic chamber of GCMiR in Katowice. The WBC treatments were performed 5 times a week (from Monday to Friday) in the afternoon (2:00 p.m.–4:00 p.m.). Each cryostimulation session was undertaken in a two-stage cryogenic chamber. During the procedure, the subjects moved slowly in a circle, one after another, without talking or touching, changing the direction of their march every 30 s. During the procedure, the participants were dressed in shorts, woolen high socks which covered the knee joints, gloves, a band or cap covering the auricles and clogs, and their noses and mouths were covered with a surgical mask containing gauze. This protocol replicated previously reported times and temperatures [32,50,51,52].

### 2.4. Measurements

The oxygen uptake (VO_2_) and respiratory exchange ratio (RER) were measured at rest (baseline) and during the SM running tests via a metabolic system (Cortex Metamax, Leipzig, Germany), and the heart rate (HR) was recorded telemetrically via an RS400 Polar-Electro (Kampele, Finland). The data were averaged over the steady-state exercise period. The internal temperature (Ti) and local skin temperatures (Tsks) were measured at rest and in the final minutes of the exercise test.

The internal body temperature was measured from the auditory canal, using an insulated, ear-molded plug and thermistor which was inserted into the external auditory canal close to the tympanic membrane and insulated from the external environment with cotton wool (Ellab, E-val-Flex model 1.38, Hillerød, Denmark). Skin temperatures were recorded during exercise on the chest (TsCh), forearm (TsF) and thigh (TsTh) via the telethermometric method (Raytek telethermometer, model 34, Poznań, Poland). Blood pressure, both systolic (SDP) and diastolic (DBP), was measured from the upper right arm via a clinically validated automatic blood pressure monitor, an Omron 711 Automatic IS (Omron Matsusaka Co. Ltd., Matsusaka, Japan). The mean weighted skin temperature (MTsk) and mean body temperature (Tb) were calculated according to Burton’s formula [53] and Stolwijk and Hardy’s equation [54], respectively. The mean arterial pressure (MAP) was calculated as follows: MAP = DBP + 1/3 × SBP. The internal-temperature-to-skin-temperature gradient was calculated as follows: Δg = Ti − skin temperature (MTsk). To determine physiological strain during exercise in the A and B exercise tests, a physiological strain index (PSI) was calculated using the equation derived from Moran et al. [55]. The PSI reflects the combination of cardiovascular and thermoregulatory strains on a universal scale; therefore, the fractional contributions of the cardiovascular (ƒHR PSI) and thermoregulatory (ƒTi PSI) systems to physiological strain were calculated according to Pokora and Zebrowska [56].

**Figure 1:**
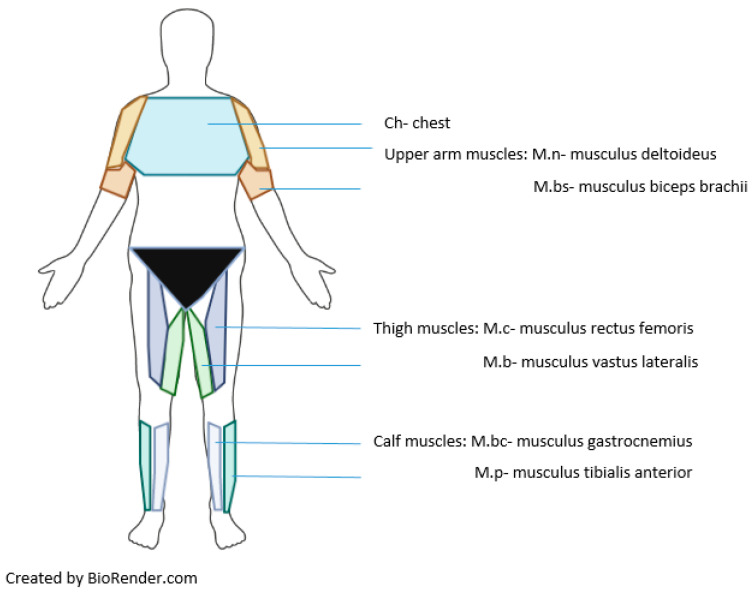
Schema presenting the parts of the body in which the average superficial temperature recorded by the thermographic camera was analyzed. Legend: Ch—chest; M.n—musculus deltoideus; M.bs musculus biceps brachii; M.c—musculus rectus femoris; M.b—musculus vastus lateralis; M.bc—musculus gastrocnemius; M.p—musculus tibialis anterior. Similar counts of pixels were detected on the left and right sides over the selected muscle zones. Ch—chest (number of pixels; 11645); M.bs, (1080); M.n, (2127); M.c, (3344); M.b, (2448); M.bc, (2329); M.p, (2362).

In order to increase the accuracy of the evaluation of skin temperature over selected surface areas and the identification of skin temperature symmetry between the R and L muscle zones before and after exercise, the thermal image method (IRT) was used. Thermal images were recorded according to the Glamorgan protocol [57], taking into account a checklist aimed at standardizing thermographic imaging in sports and exercise medicine [58], focusing on selected muscle zones according to the model proposed by Fernandez-Cuevas et al. [59]. In the IRT method, nine regions of interest on the body were selected for studying the skin temperature. The surface areas corresponded to the front of the body (the torso, right and left arms, right and left thighs and right and left calves) (Figure 1). Thermal images of the upper and lower body were taken for each participant before exercise (T0), after exercise (2 min, T1) and after 10 min of recovery (T2), using an FLIR Systems E60 camera with a resolution of 320 × 240 pixels and sensitivity of 0.05 K. The camera was calibrated with a black body, and the emissivity was set to 0.97–0.98. During imaging, the camera was positioned perpendicular to the region of interest at a distance of about 1 m from the subject. All thermographic images were analyzed by the same examiner using FLIR ResearchIR 4 Max Software (FLIR Systems, Inc., Niceville, FL, USA). This software is a tool for calculating the mean Tsk in a defined area by averaging the detected temperature values. The average temperature was analyzed on the front of the trunk and the upper and lower limbs, according to the schema in Figure 1.

Blood was collected at rest (before (A) and after (B) a series of ten WBC treatments). Blood samples were collected from the antecubital vein into the vacutainer tubes containing EDTA K. These blood samples were analyzed at a diagnostic laboratory (Katowice, Poland) within 48 h of collection, using a Sysmex XT (Sysmex K-4500, Kobe, Japan) to determine the hemoglobin concentration (Hb) and hematocrit (HCT). The other portion was assayed for analyses of the plasma catecholamines, epinephrine (Epi), norepinephrine (NE) and cortisol (CORT), via a radioimmunoassay at a certified diagnostic laboratory ANCHEM in Katowice, Poland (Automatic Gamma Counter, LKD Wallac Oy, Turku, Finland). The diagnostic kits used for the determinations were as follows: for RIA/cortisol, an RIA KIT, No. RK-240 CT, cortisol, with a sensitivity of 2.9 nmol/L and a range of 0–1600 nmol/L (0–580 ng/mL); for epinephrine, an RIA KIT, No. 3-CAT, epinephrine, with a sensitivity in plasma of 19 pg/mL and a range of 1–200 ng/mL; and for norepinephrine, an RIA KIT, No. 3-CAT, norepinephrine, with a sensitivity in plasma of 42 pg/mL and a range of 5–1000 ng/mL (Nordhorn, Germany).

### 2.5. Statistical Analysis

The distribution of the results for the analyzed variables was checked via the Shapiro–Wilk test, while the equality of variance was verified using the Levene test and sphericity was assessed using Mauchly’s test. For single measurements, the significance of differences between groups was assessed using Student’s *t*-test for dependent samples. The Wilcoxon signed-rank test was applied in the case of variables with other types of distributions, assuming statistically significant differences to be at the level of *p* < 0.05. All analyses were performed using the Statistica software package for Windows® (version 13.3, StatSoft, Kraków, Poland) program. Comparing the impact of WBC treatments on changes in the analyzed variables, an analysis of variance (ANOVA) with repeated measures was implemented to examine the influences of the main factors, i.e., exercise (SM), the whole-body cryostimulation (WBC) treatments and the SM × WBC interaction. The clinical effect size for the ANOVA was calculated using partial eta squared (η2). If a significant interaction between factors was found, Bonferroni comparisons were conducted in the post hoc analysis. For non-parametric data, Friedman’s two-way ANOVA and the Wilcoxon test were used to detect differences over time. Correlation coefficients were calculated using Spearman’s test to describe the relationships between changes in selected somatic and physiological parameters. Data with normal distributions were presented as mean values and standard deviations (x ± SDs), while the data that deviated from a normal distribution were interpreted as medians (Mes) and quartile deviations (QDs).

## 3. Results

### 3.1. The Basic Characteristics of the Study Participants were Presented in Table 1

The series of ten WBC sessions did not significantly affect either the initial physiological variables, such as the heart rate (HR), systolic (SBP), diastolic (DBP) and mean (MAP) arterial blood pressures, oxygen uptake (VO_2_), metabolic rate (M), internal (Ti) and mean body (Tb) temperatures or body mass. The results show that the subjects’ body fat significantly decreased (FM − 0.83 ± 0.96 kg, *p* = 0.01) after a series of ten WBC sessions (Table 1). The effect of the WBC interventions on physiological functions at rest was only noted for the MTsk and the internal-to-mean skin temperature gradient (Δg) (Table 3 and Table 4).

Legend: Data are shown as means ± SDs. HR—heart rate; VO_2_—oxygen uptake; rest—resting value; mean—mean value during exercise. A two-way analysis of variance (ANOVA) with repeated measures and a one-way analysis of variance (%VO_2_max) and Bonferroni’s post-hoc test were used to identify differences between A and B.

After a series of ten WBC sessions, small changes in the concentrations of stress hormones were noted at rest. We found that after a series of ten WBC sessions, the cortisol and epinephrine (EPI) concentrations (*p* = 0.37) were lower at rest, whereas the norepinephrine (NE) concentration was similar in both conditions (Table 5).

### 3.2. The Effect of WBC on Physiological Responses to Exercise

During both exercise tests (before and after a series of WBC), the mean relative exercise intensities were similar across SMA and SMB. Expressed as %VO_2_max, it corresponded to a %VO_2_max of 71.6 ± 10.9 % for SMA and a %VO_2_max of 72.0 ± 5.98 for SMB (*p* < 0.05) (Table 2).

**Figure 2:**
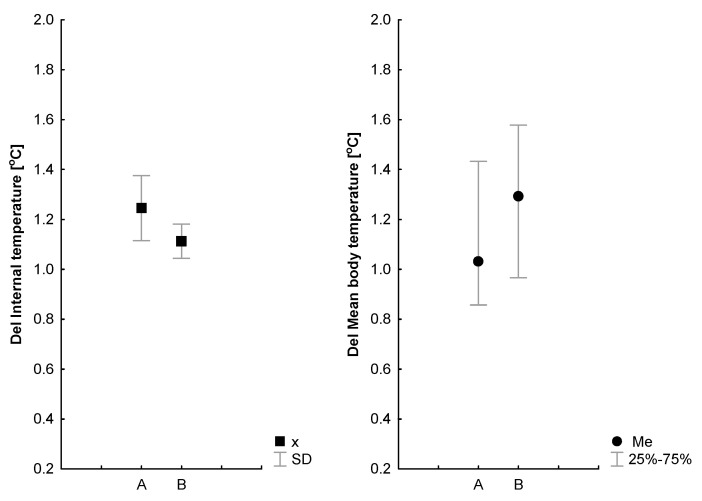
Exercise-induced changes in internal temperature (Del) and mean body temperature for the control (A) and after a series of ten WBC sessions (B). A non-parametric Wilcoxon test was used to identify differences between mean body temperatures. Values are presented as medians (25–75%).

There was no main effect of the cold thermal interventions on internal temperature Ti (*p* = 0.11). The main effect of exercise was proven for the Ti (*p* = 0.00, η2 = 0.58), but there was no evidence of an interaction effect (Table 3). The Ti increased during exercise in all subjects, but the end (Ti_last_) temperature recorded during an exercise session was higher in A and B (*p* = 0.02) (Table 4). The average internal temperature, ΔTi, during exercise tended to be lower after WBC (*p* = 0.6) (Figure 2).

There was no main effect of WBC for the mean body temperature (Tb) (*p* = 0.12). The baseline Tb was similar during measurements A (34.6 ± 0.4 °C) and B (34.8 ± 0.7 °C) (*p* > 0.05). There was an effect of exercise (*p* = 0.00, η2 = 0.7), but no evidence of an interaction effect for the ΔTb (*p* = 0.12). The last Tb temperature elicited in an exercise session was similar for A and B (Table 4). The average ΔTb during exercise tended to be higher after WBC (*p* = 0.6) (Figure 2).

There was a main effect of the treatments for the mean skin temperature (MTsk) (*p* = 0.003, η2 = 0.57) and an effect of exercise (*p* = 0.001, η2 = 0.8), but no interaction effect (*p* = 0.34) for the MTsk. The MTsk measured during the last exercise workload was higher in B than in A (~0.7 °C) (*p* = 0.001).

There was no main effect of the WBC procedures on the participants’ systolic (*p* = 0.9) and diastolic (*p* = 0.4) blood pressures. There was an effect of exercise (*p* = 0.005, η2 = 0.3) on the SBP and (*p* = 0.049, η2 = 0.2) DBP, but no evidence of an interaction effect (Table 4).

There was no main effect of the WBC procedures on the metabolic rate (M) (*p* = 0.6). There was an effect of exercise (*p* = 0.00, η2 = 0.3), but no evidence of an interaction effect for the M (*p* = 0.3) (Table 4).

There was a main effect of the WBC sessions on the temperature gradient (Δg = Ti − MTsk) (*p* = 0.00, η2 = 0.7) and an effect of exercise (*p* = 0.02, η2 = 0.2), but no evidence of an interaction effect. The Δg = Ti − MTsk value, measured before exercise and during the last exercise workload, was significantly lower for B than for A (Table 4).

There was no main effect of the WBC sessions on the EPI, NE and CORT, but there was an effect of exercise on all the hormones tested (EPI: *p* = 0.000, η2 = 0.58; NE: *p* = 0.001; η2 = 0.9; and CORT: *p* = 0.003 η2 = 0.3) (Table 5). An interaction effect (intervention x exercise) was evidenced only for CORT (Table 5).

Significant PSI differences were found for the matched experimental model between sessions A and B (*p* = 0.038, ES = 0.87). No significant differences were found for the ƒHR PSI and ƒTi PSI between A and B (Table 4).

### 3.3. The Effect of WBC and Exercise on Skin Temperature Behavior following Exercise

**Figure 3:**
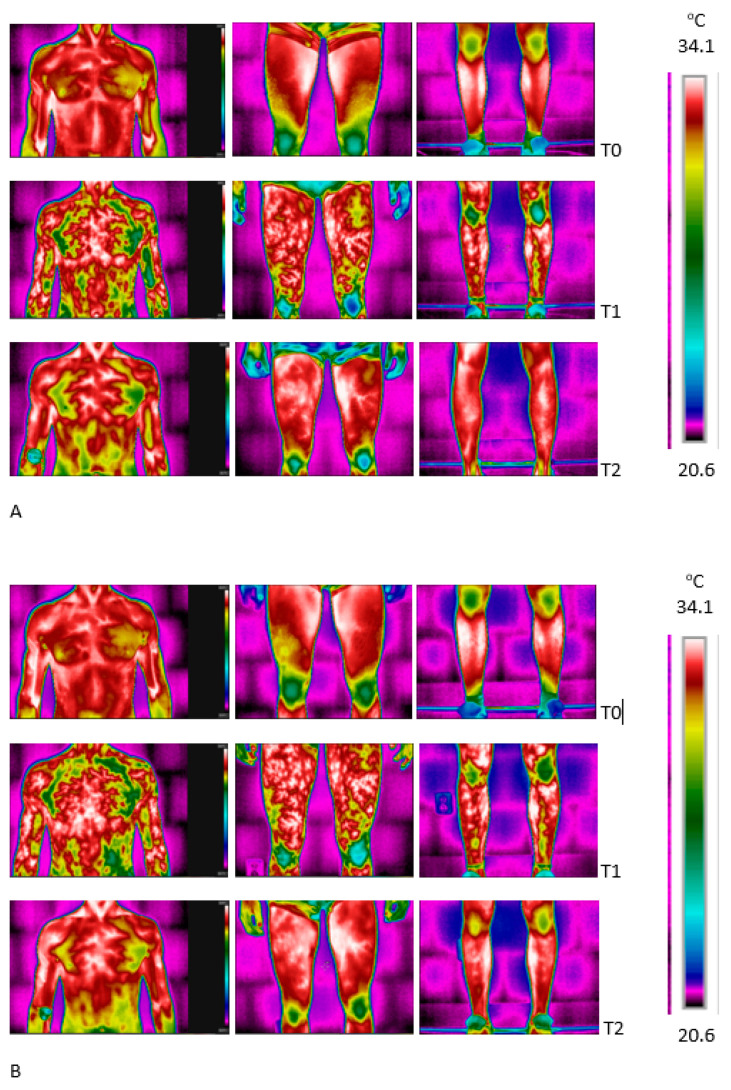
Sample ROIs analyzed before and after exercise (SM) and after 10 min of recovery in control (**A**) and after a series of ten cryostimulation sessions (**B**). Note: T0—rest; T1—after the end of the exercise test; T2—after 10 min of recovery.

There was a difference between the skin temperatures registered at rest via the telethermometric method and as thermal images of the upper- and lower-body surface areas (Figure 3).

**Figure 4:**
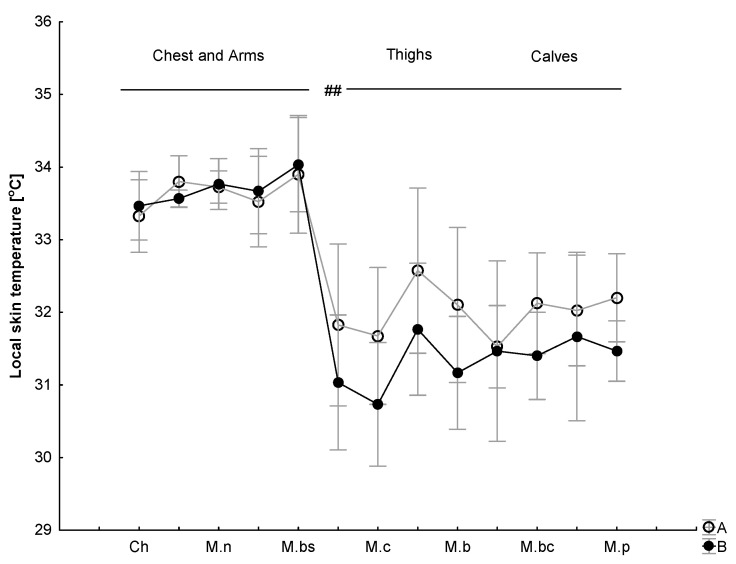
Skin temperature (°C) distribution at rest (chest, arms, thighs and calves) for the control (A) and after a series of ten WBC sessions (B). ## *p* ≤ 0.01 indicates a significant difference between the regions of the thighs, calves and chest and arms. Legend: Ch—chest; M.n—musculus deltoideus; M.bs—musculus biceps brachii; M.c—musculus rectus femoris; M.b—musculus vastus lateralis; M.bc—musculus gastrocnemi-us; M.*p*—musculus tibialis anterior.

The baseline local skin temperature of the chest and arms was 1.5–2.0 °C warmer than the thighs and calves. After the WBC exposure, similar site-specific differences were observed between the regions of the thighs, calves and arms (Figure 4).

**Figure 5:**
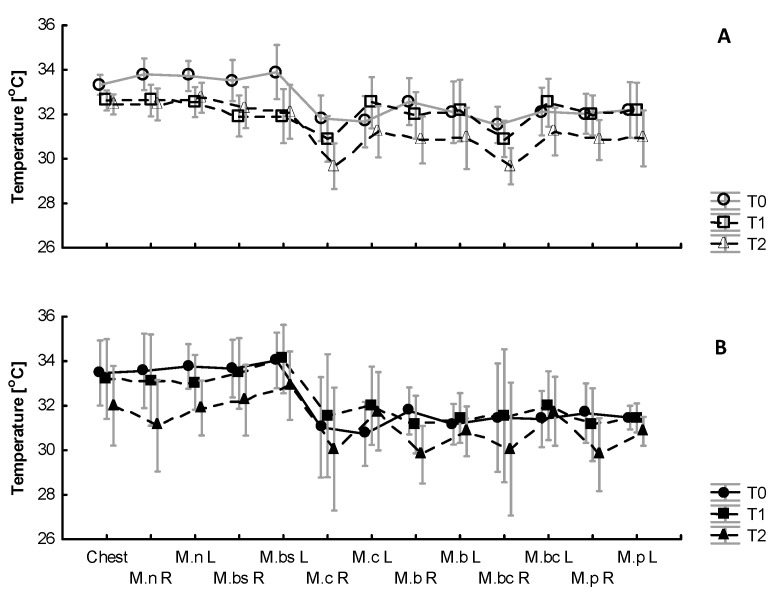
The results of the thermograms analyzed for the chest, arms and lower limbs (ROIs) before (**A**) and after (**B**) a series of ten cryostimulation sessions. Legend: M.n R—right musculus deltoideus; M.n L—left musculus deltoideus; M.bs R—right musculus biceps brachii; Mbs L—left musculus biceps brachii; M.c R—right musculus rectus femoris; M.c L—left musculus rectus femoris; M.b R—right musculus vastus lateralis; M.b L—left musculus vastus lateralis; M.bc R—right musculus gastrocnemius; M.bc L—left musculus gastrocnemius; M.p R—right musculus tibialis anterior; M.p L—left musculus tibialis anterior; T0—at rest; T1—at 2 min after the end of the exercise test; T2—after 10 min of recovery.

At rest, there were significant differences between the skin temperatures of the upper and lower body areas before and after the WBC sessions (*p* < 0.5) but no significant difference was found in the thermal imaging between the same muscles after a series of ten WBC treatments (B) compared to the control (A). There was an effect of exercise (*p* = 0.005, η2 = 0.3) on temperature for all the tested regions of skin (*p* = 0.049, η2 = 0.3). Local changes in the skin temperature in selected surface zones in response to exercise and after 10 min of recovery are presented in Figure 5.

**Figure 6:**
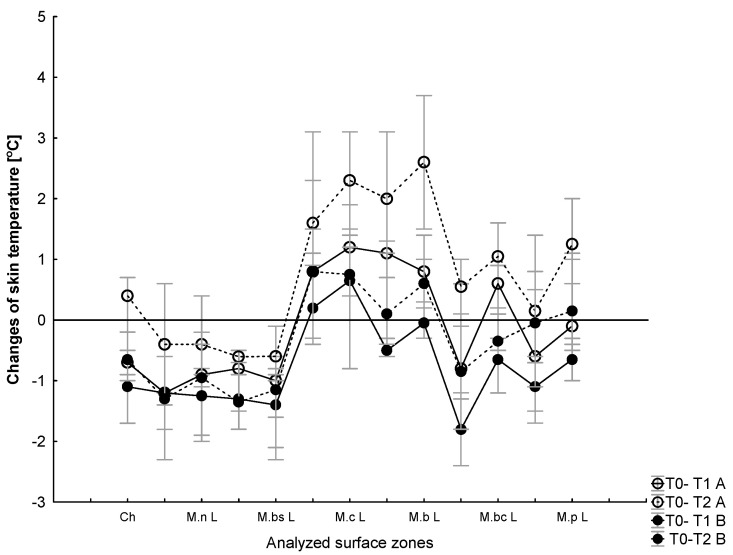
Changes (Δ) in skin temperature over the selected surface areas after exercise and after 10 minutes of recovery in comparison to the baseline (T0) for the control (A) and after the WBC treatments (B). Values are presented as medians (25–75%).

With respect to temperature increments over the selected areas, there were no significant changes in the temperature measured over the chest and arms immediately after the exercise test (T1) compared to T0 in A (*χ*2 = 2.86, *p* = 0.57) and in B (*χ*2 = 5.4, *p* = 0.17). Regarding temperature changes over the thigh muscles, there were no significant changes in the temperature measured after the exercise test (T1) compared to T0 in A (*χ*2 = 1.47, *p* = 0.69;) and in B (*χ*2 = 59.4, *p* = 0.17). Regarding temperature changes over the calf muscles, there were no significant changes in the temperature measured after the exercise test (T1) compared to T0 in A (*χ*2 = 5.03, *p* = 0.16) and in B (*χ*2 = 5.8, *p* = 0.12) (Figure 6).

Regarding changes in temperature over the tested surface areas over the chest and arms, there were significant changes in the temperature measured after 10 minutes of recovery (T2) compared to T0 in A (*χ*2 = 11.59, *p* = 0.02) but not in B (*χ*2 = 7.56, *p* = 0.11). Similarly, over the tested surface areas over the thighs, there were changes in the temperature measured after 10 minutes of recovery (T2) compared to T0 in A (*χ*2 = 7.08, *p* = 0.06) but not in B (*χ*2 = 3.81, *p* = 0.28). Regarding temperature changes over the calf muscles, there were no significant changes in the temperature measured after 10 minutes of recovery compared to T0 in A (*χ*2 = 4.39, *p* = 0.22) and in B (*χ*2 = 1.11, *p* = 0.77) (Figure 6).

There was a significant difference between moments T0 and T1 in which the Tsk decreased at T1 in comparison to T0 (−∆ 0.61 ± 0.1 °C) on the chest and (−∆ 1.40 ± 0.2 °C) on the arms in A and (−∆ 0.52 ± 0.1 °C) on the chest and (−∆ 1.23 ± 0.1 °C) on the arms in B. The chest skin temperature stayed above the resting condition after 10 min of recovery (T2) in A (∆ 0.42 ± 0.2 °C) but fell below the resting state during 10 min of recovery in B (∆ −1.1 ± 0.2 °C).

Regarding the anterior parts of the arms, there was a significant difference between moments T0 and T2 in A and B in which the Tsk stayed still below the resting state during 10 min of recovery (T2) in comparison to T0 (−∆ 0.78 ± 0.1 °C) in A and (−∆ 1.73 ± 1.50 °C) in B (Figure 6).

Regarding the leg muscles, there was a significant difference between moments T0 and T1 in which the Tsk increased at T1 in comparison to T0 (+∆ 1.53 ± 0.1 °C) in A and (+∆ 0.78 ± 0.1 °C) in B, staying above the resting condition during 10 min of recovery (T2) in A (∆ +2.06 ± 0.1 °C) and in B (∆ + 0.52 ± 0.3 °C).

There was a significant difference between moments T0 and T1 in A and B with respect to the calf muscles in which the Tsk increased at T1 in comparison to T0 and stayed above the resting state after 10 min of recovery (T2) in comparison to T0 (+∆ 0.78 ± 0.2 °C) in A and decreased below the resting state (−∆ 0.73 ± 0.3 °C) in B. None of the skin temperatures reached the reference values during the 10 min recovery period (Figure 6).

**Figure 7:**
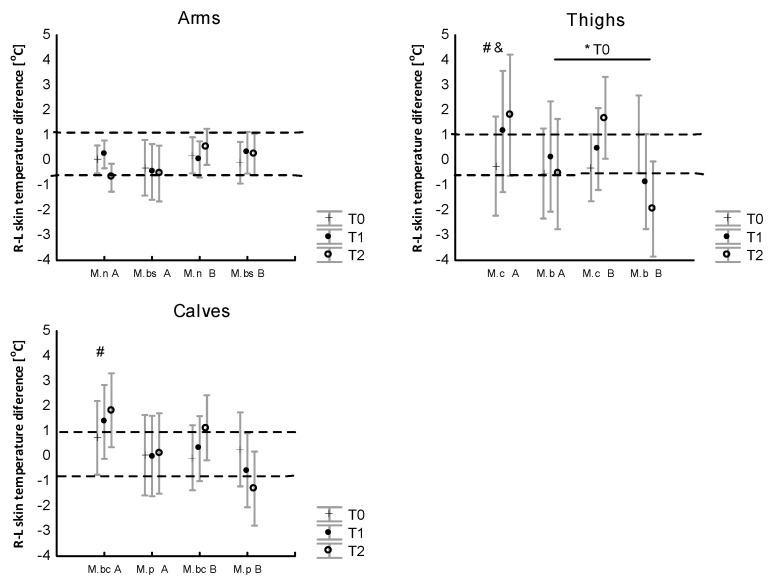
Differences in skin temperature on the R–L sides of selected muscle zones at rest (T0), after exercise (T1) and after 10 min of recovery (T2) for the control (A) and after WBC treatments (B). * *p* < 0.05 indicates a significant difference between experiments A and B; # *p* < 0.05 indicates a significant difference within the group between T0 and T1; & *p* < 0.05 indicates a significant difference within the group between T0 and T2.

In respect to the detection of asymmetry between the R and L muscles, it was found that there was no main effect of the WBC procedures for the symmetry of the bilateral surface areas of the muscles in the arms (*p* = 0.86), thighs (*p* = 0.93) and calves (*p* = 0.93). There was an effect of exercise (*p* = 0.008, η2 = 0.3) for the arm muscles (*p*= 0.0089, η2 = 0.6), thighs and calves (*p* = 0.003, η2 = 0.5), and evidence of an interaction effect only for the thigh muscles (*p* = 0.003; η2 = 0.7). Differences in skin temperature on the R–L sides of selected muscle zones at rest (T0), after exercise (T1) and after 10 min of recovery (T2) for the control (A) and after the WBC treatments are presented in Figure 7.

## 4. Discussion

The current study is the most thorough investigation of the effects of a series of ten WBC sessions on physiological responses to exercise and skin temperature behavior following exercise in athletes. The main findings of this investigation are as follows:

(1) A series of 10 cryostimulation treatments induced significant changes in body composition (FM) but did not cause changes in the participants’ body mass. After a series of 10 WBC treatments, there were no significant changes in variables such as the metabolic rate (M), the oxygen uptake (VO_2_), the heart rate (HR), the internal temperature (Ti), the systolic (SBP) or diastolic (DBP) blood pressure and the mean arterial blood pressure (MAP), but the mean skin temperature (MTsk) was significantly higher, and the temperature gradient between the internal temperature and the mean skin temperature (Ti − MTsk) was lower.

(2) The series of 10 WBC treatments did not significantly affect the resting concentrations of the stress hormones tested. The concentration of cortisol was modified by the applied WBC therapy and exercise, and after cold therapy, it was lower than before the treatments. During exercise, after a series of 10 WBC treatments, the physiological strain index (PSI), the increase in internal temperature and the skin temperature were significantly lower than before the treatments. Before and after the series of WBC treatments, local skin temperatures before exercise (measured via both the telethermometric method and using thermal imaging) were ~1.5 °C higher in the upper body than in the lower body, but no features of the asymmetry of the contralateral skin areas were noted.

(3) After the exercise, during the recovery period, a significant (contralateral) asymmetry in skin temperature between the right and the left thigh muscles was noted. A series of 10 cryostimulation treatments tended to reduce the exercise-induced temperature asymmetry over the thigh muscles.

### 4.1. Changes in Body Composition, Metabolism and Cardiovascular Function Induced by a Series of Ten WBC Treatments

The series of 10 WBC treatments did not result in a significant reduction in body mass, but a significant reduction in fat mass was noted (ΔFM; −0.83 ± 0.93 kg). The obtained results contradict the results obtained by Ziemann et al. [12] who, after a series of WBC treatments, compared the effect of the cryostimulation treatments in people with lower cardiopulmonary capacity (VO_2_max 25.7 ± 2.0 mL/kg/min, BMI 34.0 ± 2.5 kg/m^2^) and higher cardiopulmonary capacity (VO_2_max 43.3 ± 3.0 mL/kg/min, BMI 31.4 ± 2.0 kg/m^2^) and did not notice changes. Also, Lubkowska et al. [9] found no significant changes in body composition following the application of a series of WBC treatments despite supporting the treatments with physical training. The results, which indicate a decrease in %fat, are consistent with the results of Pilch et al. [60], who found that in a study of younger men with low levels of physical activity, men with both obese and normal body BMI values recorded significant decreases in body mass and the fat content after a series of 20 WBC treatments (2–3 min, −120 °C). The subjects in our group were characterized by high levels of physical fitness (VO_2_max > 60 mL/kg/min) and low BMI values. Obviously, the effectiveness of WBC treatments capable of reducing body weight and affecting body fat content depends on the degree of the respondents’ obesity, their baseline BMI, their gender and age and the number of treatments [61,62,63]. After 10 WBC sessions, Więcek et al. [63] reported significant reductions in the waist, hip and abdominal circumferences, waist-to-height ratio, triceps and abdominal skinfold thickness in one group of women with overweight and two groups of menopausal women with obesity. After 20 sessions of WBC, these effects intensified [63]. It can be assumed that in the case of lean, young athletes, somatic changes involving a reduction in body fat (changes in body composition) could be observed earlier, after a series of 10 treatments.

There is recognition that cold therapies could support changes in body composition via increased thermogenesis, the activity of brown adipose tissue and subsequent caloric burn and fat loss [64]. However, it is noteworthy that individuals with a higher fat content may maintain core and tissue temperatures to a greater extent after cryotherapy (due to reduced vasodilation) compared to leaner individuals [29]. Additionally, subcutaneous adipose tissue provides thermal insulation and reduces thermal conductivity [30]. Higher amounts of subcutaneous fat in individuals with obesity could cause lower heat loss [65] and slow the increase in the metabolic rate compared to individuals without obesity. Consequently, the response and overall tolerance to low temperatures may differ accordingly in lean and obese individuals. Although several studies suggest that WBC leads to reductions in body fat and circumference in different regions of the body, to date, there is a limited body of evidence regarding its efficacy in mobilizing body fat in athletes, and empirical studies detailing the potential mechanism(s) through which this therapy may be effective in fat reduction remain under-investigated.

Exposure to cold which is accompanied (or not) by shivering thermogenesis may increase the rates of metabolic processes, which often manifests as an increase in the basic metabolism [66,67]. The results obtained in this study did not show significant changes in the rates of metabolic processes at rest after a series of 10 WBC treatments. The results of our research are consistent with previous research in which no changes were observed or the observed increase in the metabolic rate after a series of cryostimulation was small (~20.83 kcal/h) when compared to the results of an examination prior to the treatments [68]. Leppäluoto et al. [32], who studied a daily 2 h exposure to cold air at 10 °C for 11 days, believe that the cold-induced stimulation of the metabolic rate is attenuated after repeated cold exposures due to adaptation to cold, but the temperatures and acclimation time in this study were different [32,68] than in our study.

An analysis of the study results indicated that a series of ten 3 min exposures of the body to a cryogenic thermal agent with a temperature of approx. −110 °C does not cause significant changes in the heart rate (HR), systolic blood pressure (SBP), diastolic blood pressure (DBP) or mean arterial blood pressure (MAP) compared to the measurements of these variables at rest before treatment (A). They were also not accompanied by significant changes in the concentrations of stress hormones in the blood. In the current study, there were no significant changes in the concentration of noradrenaline after acclimation to cold, although the level of adrenaline was lower in thermoneutral conditions after a series of WBC treatments, which is in line with the trend observed in the research by Van Der Lans et al. [68]. The lack of changes involving the HR or SBP and DBP after a series of ten WBC treatments is consistent with the results presented by Lubkowska and Szyguła [69]. In their study, each session caused a significant increase in both systolic and diastolic blood pressure after each cryostimulation which was accompanied by a significant decrease in the heart rate (by about seven beats per minute). However, these changes did not persist after the stressor stopped acting. Changes in the ΔSBP, ΔDBP and ΔMAP were not different on the 1st, 5th, 10th and 15th days of the experiment. Based on these results, the researchers found that there were no adaptive changes involving blood pressure and heart rate in response to repeated cold stress [69]. In another study, Klimek et al. [52] applied a series of ten 3 min exposures to cryogenic temperatures (cryostimulation in men and women), similar to the current study, they did not note any significant changes in the heart rate and blood pressure after the series of treatments. Repeated exposures to WBC were mostly tolerated well by the participants, who became accustomed to the stressor at an early stage of the treatment [28], and the increase in blood pressure that is usually observed after cryostimulation is short-lived and characterized by high individual variability within the study group.

Habituation is the most commonly observed pattern of thermoregulatory adjustment observed in response to chronic or repeated cold exposure. As habituation develops, physiological responses to cold become less pronounced (blunted shivering, blunted cutaneous vasoconstrictor response or both) than in the unacclimated state [30,32,70]. The achieved adaptive changes to cold can affect the somatic and functional characteristics of the body at rest and shape the characteristics of temperature, physiological, metabolic or immune responses to physical effort [9]. Remie et al. [70] believe that prolonged exposure to moderate or mild cold stress may lead to weaker defensive responses against cold and weaker or later experienced exercise stress due to the development of habituation to cold [70]. Since, physical exercise causes an increase in the activity of the sympathetic system depending on the intensity of the exercise, actions that can significantly inhibit the volume of the body’s response to exercise stress (by quickly restoring parasympathetic activity), thus contributing to the post-exercise recovery process, which is an important reason for the use of a series of whole-body cryostimulation treatments in sports, are desirable.

Studies have shown that a series of WBC treatments has a strong effect on skin temperature at rest but does not cause significant changes in internal temperature [28]. Our results are consistent with those observed in the study by Leppäluoto [32], who noted a higher skin temperature after a series of WBC treatments which was not accompanied by a significant change in internal temperature. Adipose tissue is considered a potential influencing factor on global and local skin temperature (Tsk) values [46,68,71,72,73], but in the current study, the increase in the MTsk (ΔMTsk: +0.89 ± 0.52 °C) showed no association with a reduction in the FM% (Me; QD) (ΔFM: −0.7; −2.85, −0.5%) r = 0.17 or FM (ΔFM: −0.6; −2.2, −0.2 kg) r = 0.12 after a series of ten WBC treatments. An elevated skin temperature after adaptation to cold, combined with a decrease in the vasoconstrictive response to cold, may be an element of cold habituation. A higher (although not significantly) skin temperature at rest after a series of cold treatments was observed in studies by van Der Lans et al. [68] under thermoneutral conditions. In other studies which evaluated changes in skin circulation, it was noted that the regular use of cold treatments may induce changes in angio-motor control, as well as the sensitivity of skin vessels to the stressor and the action of hormones [74]. However, the results regarding the distribution of surface temperatures after whole-body cryostimulation remain inconclusive [2]. Individual body parts react differently to extremely low temperatures, and heat loss is not identical in all regions and depends on the thickness of skin folds [50,75,76,77], the local percentage of fatty tissue [46], muscle mass [75] and the sport level [78,79,80]. The above features cause different degrees of effectiveness when inducing changes in skin temperature in persons subjected to cryogenic stimulation.

Immediately after the session, the cooled areas are heated by an influx of warm blood. It is a reflex and an elementary mechanism of thermoregulation after cryostimulation sessions. Szygula et al. [81] proved significantly greater active skin hyperemia following a decrease in pO_2_ and the accumulation of metabolites, which was similar in women and men, after a series of 10 cryostimulation treatments. According to Leppäluoto et al. [32], the application of extremely low temperatures may significantly affect the myogenic control of blood flow. Stanek et al. [82] demonstrated the beneficial effects of WBC treatments on endothelial function following 10 daily WBC exposures (3 min; −120 °C) followed by kinesiotherapy. This improvement in endothelial function, achieved in healthy, 40-year-old men, was manifested via a reduction in inflammatory markers, increased antioxidant defense as a result of WBC and the protective effects of the increased bioavailability of NO as a result of an increased level of iNOS. iNOS is up-regulated after a series of cryostimulation treatments, as demonstrated in an animal model [83] and in human studies [84]. It can therefore be hypothesized that the increase in the blood iNOS concentration obtained after a series of WBC treatments [84] may exert a beneficial effect on skin circulation, increasing the bioavailability of NO and the operation of this mechanism, modulating the tone of vascular smooth muscle in the skin and the skin temperature.

After a series of WBC treatments, the skin temperature at rest was higher than before the application of the treatment. Considering the fact that after experiencing stimulation with cold, a decrease in sympathetic stimulation occurs as a result of adaptation to the cold, and that increased skin hyperemia can be sustained by an increased release of NO [85,86], which is greater after a series of cryogenic treatments [84], it can be assumed that the observed higher MTsk values at rest after a series of WBC treatments in athletes seem to reflect the occurrence of adaptive changes in skin circulation after repeated exposure to cold.

Under environmental conditions close to thermal neutrality, the temperatures on the surface of the body surface vary greatly. In examinations at rest, conducted both via telethermometric measurements and in thermal imaging, differences in the skin temperatures of the upper- and lower-body areas typical of the conditions of moderate ambient temperature were noted. Under ambient temperature conditions of 21 °C, the differences in the skin temperatures of the upper- and lower-body surfaces reached approx. 1.5 °C. This area variation in skin temperature was observed both before and after a series of WBC treatments. It is worth mentioning that cutaneous blood flow varies in different regions of the body, mainly due to local differences in the control of the tone of the vascular smooth muscles in the skin, with large differences between apical and non-apical regions [87,88], as noted in the current research.

### 4.2. Changes in Metabolism, Cardiovascular Function and the Body’s Temperature in Response to Exercise after a Series of Ten WBC Treatments

In the current study, the mean power (W/kg bw) and exercise intensity, expressed as %VO_2_max, during the test effort were similar before and after 10 WBC sessions. The applied series of 10 cryostimulation treatments did not have a significant impact on the metabolic rate (M), blood pressure values (SBP, DBP and the MAP) in response to physical effort. However, it was noted that the WBC series substantially affected the mean weighted skin temperature (MTsk) at rest and induced changes in the Ti and MTsk, as well as local skin temperatures during and after the exercise test. Most of the research on the evaluation of thermoregulatory function conducted during a running effort [89,90,91] focused on the analysis of two distinct temperatures: the internal temperature (Ti) and skin temperature (Tsk). The internal temperature is centrally regulated [91], is maintained at a relatively constant level of 37 °C at rest and increases during exercise as a result of an increased metabolic rate and metabolic heat production; it and is largely independent of environmental conditions [89,90].

The results of the current study show that a series of WBC treatments significantly affected local skin temperatures, the increase in internal temperature and changes in local skin temperatures in response to physical exercise. The exertional increase in the Ti was smaller after the WBC series than in the control (1.24 °C vs. 1.1 °C). Considering that an increase in internal temperature reflects changes in the balance between the rates of storing and dissipating heat from the body [90], a smaller increase in the Ti during exercise with a similar metabolic rate of heat production during the test after the WBC series shown in the study may indicate efficient heat dissipation in both sessions of the experiment; however, in the group of respondents using a series of whole-body cryostimulation treatments, the thermal deformation (defined as the level of deviation of the Ti from the resting value) was smaller. During exercise, there was a lower physiological stress, assessed as the PSI index (*p* = 0.04), in subjects after the WBC series. The PSI index reflects the combined loads of the circulatory system and thermoregulation, with both parameters having an equal share in the physiological load. The PSI index was calculated using changes in the internal temperature (Ti) and heart rate (HR), according to Moran et al. [55]. This method combines measures of the cardiovascular load (ƒHR PSI) and thermoregulation (ƒTi PSI) [56]. The results of the current study showed that during exercise, the PSI index and ΔTi were lower after the WBC series (B) than in study A, indicating that the reduction in the thermal load after a series of WBC treatments contributed to lower physiological stress during exercise. In addition, higher MTsk temperatures at rest and smaller changes in the ΔMTsk during and after exercise were noted. This may have resulted from a weakened/suppressed blood vessel response to the exercise stimulus after the series of 10 WBC treatments, which may have been a consequence of changes in myogenic blood flow control [32] and decreased sympathetic activity or a reduction in the sensitivity of adrenergic receptors, which may lead to vasodilation or vasoconstriction during exercise [4,30] after a series of WBC treatments. Ultimately, the above changes could modulate the convective transfer of heat from the body’s core to the perimeter and affect the increase in the skin temperature over active muscles in response to exercise and after its completion, as observed in the current study.

Interestingly, after exercise, the skin temperature increased in the front of the thigh, while it decreased in the front of the chest. After a detailed analysis of ROI thermographic images, these results were found in both trial A and trial B. Changes in skin temperature may reflect changes in the blood supply to selected skin areas in response to exercise [43]. Exercising can be accompanied by a varied skin temperature response which depends on the type of exercise, its nature and the volume of the muscle groups involved [92]. Periodic changes in the distribution of skin surface temperatures result from the fact that during exercise, there is a redistribution of blood flow from inactive areas, such as visceral fat, skin and muscles not recruited during the exercise, to active skeletal muscles through the control of vasodilation and vasoconstriction [93,94,95].

### 4.3. Skin Surface Temperature Behavior (IRT) following Exercise Test under Thermoneutral Conditions after a Series of Ten WBCs

The running effort that was applied in the current study promotes a large and intensive stimulation of the rectus femoris, the vastus medialis and the vastus intermedius muscles [96,97]. The distribution of skin temperatures recorded in the ROI images before and after the period of acclimation to cold (a series of WBC treatments) was not significantly altered at rest and was characterized by a constant value in the range of 32–34 °C. However, a characteristic −1.5 °C difference in the skin temperatures of the upper- and lower-body regions was noted. In response to exercise (the second minute of recovery), the skin temperature above the chest clearly dropped, which seems to be a consequence of increased evaporative cooling caused by sweating rather than a vasoconstriction response in this area of the skin. On the other hand, the skin temperature rose on the surface of the thigh after the exercise and hyperthermic spots were clearly visible, which was probably associated with dilation of the cutaneous vessels occurring after exertion.

In the case of exercises performed with constant loads, the skin temperature at the beginning of work usually drops and then rises over time. The initial decrease and subsequent slight increase in the skin temperature is thought to be the net result of a combination of vasoconstriction and vasodilatation responses [43]. The results of this research showed a varied area of skin thermal responses in each ROI due to the variation in the involvement of different body segments in work while exercising. Immediately after the exercise, the Tsk increased in the quadriceps and calf muscles, while the Tsk decreased in the chest and shoulders. Specific differences were observed in the Tsk response after exercise that were dependent on the previously applied (or not) thermal intervention. The differences were manifested in a greater amplitude of changes in skin temperatures over inactive muscles and a smaller increase in temperature over the active muscles of the lower limbs immediately post-exercise in the group subjected to a series of WBC treatments. There was also a variation in the time of the occurrence of the largest changes in skin temperature after exercise (after 2 min vs. 10 min of recovery) and in the absolute increases in skin temperature. The greatest reduction in skin temperature after exercise was recorded on the surface of the chest and shoulder muscles. A decrease in skin temperature above the muscles of the lower limbs was recorded only in the 10th minute of post-effort recovery following the WBC intervention (B) and much earlier, in the 2nd minute of recovery, in study A. A greater delay in the onset of post-exercise Tsk changes over active skeletal muscles after the WBC series could be a consequence of changes in the reactivity of the skin vessels to the exercise stimulus, which could affect the extent of the vasodilation/vasoconstriction of the cutaneous vessels during exercise and affect the behavior of the blood flow in the skin and skin temperature after exercise.

Vasomotor control primarily depends on internal temperature, while the Tsk exerts its effect via (1) direct local action on blood vessels, (2) chemical action or (3) axon reflexes. Considering the above, it can be assumed that the Tsk behavior on the surfaces of the lower limbs may manifest different features of temperature regulation compared to the upper body. The results of this research proved a clear increase in the temperature of the skin surface over the muscles involved in running. A thermogram analysis revealed that a greater drop in skin temperature was noted immediately after exercise, and it concerned areas that were less involved in the exercise work. A similar decrease in temperature in all analyzed body areas was recorded immediately after intense effort [95,98], while in a study by Tanda [36], significant differences in skin temperature were observed over areas located above the groups of muscles which were active or non-active during exercise. The variation in skin temperature depending on the location (over active vs. inactive muscle groups during exercise) may support the statement that this effect could be associated with a different load on the musculoskeletal system, a difference in the efficiency of heat dissipation during exercise and the metabolic production of heat which occurs in working muscles compared to inactive muscles [75,94,95,99,100].

It is worth adding that at rest, both before and after a series of whole-body cryostimulation treatments, the athletes were characterized by a temperature symmetry of bilateral skin areas which was within the reference values for healthy people (<1 °C) [101]. In physiological conditions, in the absence of disorders in the musculoskeletal system and changes related to inflammation, the human body is thermally symmetrical [102,103]. In this study, no asymmetry was found in either the mean or individual temperature values between the analyzed areas of the right and the left sides of the body at rest in thermally comfortable conditions. However, a detailed analysis of changes in skin temperatures after physical effort showed a temporary increase in the temperature difference between the right and the left surfaces of the lower limbs, amounting to >0.5 °C and hence considered physiologically significant. Therefore, following the running effort, a significant temperature asymmetry occurred, especially over the areas of the lower limb muscles (active in the running effort) which was maintained during the recovery period. The application of the series of WBC treatments did not significantly reduce the temperature asymmetry in the examined areas, although a trend in such changes was noted over the thigh muscles.

The assessment of bilateral asymmetry is used to determine the potential risk of injury and functional deficits in athletes and non-athletes [104,105], as well as to quantify the motor performance of healthy individuals and athletes [106,107,108,109,110]. The post-exercise temperature asymmetry observed in the current study, although higher than the reference values for healthy subjects, appears to be a physiological response to exercise. Recently, assessments of the legs and hips [79,111] have recorded changes in skin temperature of above 1 °C in the contralateral part after physical exertion. Likewise, Knyszyńska et al. [98] saw an increase in the post-exercise temperature asymmetry in a group of swimmers. Interestingly, when assessing bilateral changes in skin temperature using the IRT method after symmetrical and asymmetrical exercises in professional rowers and handball players, the authors of [112] proved that the bilateral post-exercise skin temperatures of the symmetrical front and rear sides of the arm, forearm, thigh and trunk exhibit features of temperature asymmetry which, however, fall within the range of normal values. Although it is believed that a temperature asymmetry of >1 °C is associated with skeletal muscle dysfunction, the presence of thermal asymmetry may also result from prolonged activity of the sympathetic nervous system and individual differences in sympathetic regulation and may be associated with changes in muscle performance [113], which is an element of the changes accompanying muscle work. The reported changes in the Tsk in response to exercise may therefore have resulted in a decrease in the temperature asymmetry of the lower limb muscles following exercise performed after a series of ten WBC sessions in elite athletes.

The results of the current study indicate that the application of a series of ten WBC stimulations during the TP phase in cross-country skiers may modify the body composition of the athletes, although it does not lead to significant functional changes that are revealed at rest. Apart from this, a series of 10 WBC treatments can significantly reduce overall physiological stress (the cardiovascular and thermoregulatory systems) during moderate-intensity exercise and promote better maintenance of the temperature symmetry of the lower limb muscles, which indirectly indicates a lower risk of heat- and exercise-induced muscle damage (EIMD).

This study indirectly reinforces the importance of considering the use interventions such as WBC in sports and exercise medicine to improve athletes’ wellbeing and performance, emphasising the potential clinical applications of such treatments in sport and physiotherapy.

### 4.4. Limitations

The main limitation of this study is the relatively low number of participants in the study group due to the scarcity of elite cross-country skiers of an appropriate performance level who were willing to qualify for the study. Another limitation is the fact that the transition period of the annual training plan was not purely an adaptation to cold as it combined physical activity with cryostimulation exposure. Therefore, we cannot rule out the possible covariance of cold and physical activity. In addition, the exercise test was performed under thermoneutral conditions, and responses may vary in hot and cold conditions; therefore, the results discussed above are limited to the realms of the thermoneutral conditions used in this study.

## 5. Conclusions

The results of the research conducted show that the use of a series of 10 whole-body cryostimulation treatments causes noticeable changes in the amount of body fat; however, it is not accompanied by significant changes in body weight, the metabolic rate or cardiovascular function. In addition, it was found that the applied series of cryogenic temperatures reduced the thermal load and physiological stress during exercise and variously affected the skin temperature and its changes in response to and after exercise. The magnitudes of the changes observed in skin temperatures varied and depended on the skin surface areas tested. The above observations allow for the conclusion that a series of ten cryostimulation treatments significantly affects the body’s thermal reactions in response to physical exercise performed in thermoneutral environmental conditions by professional cross-country skiers; these reactions manifest as the suppression of the reactivity of the skin’s blood vessels to exercise stimulus, a change in the distribution of blood flow in cutaneous circulation and in the share of selected areas of the skin in the dissipation of excess heat generated during muscle work. However, a more extensive study must be performed to confirm our results.

## Figures and Tables

**Table 1:** The study participants’ somatic and aerobic fitness characteristics.

Indicators		
N = 17	x ± SD	
Age	21.5 ± 2.2	
Height (cm)	179.5 ± 5.7	
Body mass (kg)	75.18 ± 5.8 ^A^	74.91 ± 5.9 ^B^
Body mass index (kg·m^−2^)	22.05 ± 1.9	
Fat mass (kg)	7.78 ± 1.7 ^A^	6.94 ± 1.9 ^B^*
Fat mass (%)	10.09 ± 2.2 ^A^	9.31 ± 2.6 ^B^
Fat-free mass (kg)	67.4 ± 5.6 ^A^	67.9 ± 6.1 ^B^
Body surface area (m^2^)	1.93 ± 0.1	
Training status (years)	9.2 ± 10.5	
Power_max_ (W)	395.7 ± 9.1	
VO_2max_ (ml·kg^−1^·min^−1^)	67.5 ± 6.4	
HR_max_ (bs·min^−1^)	191.5 ± 12.8	
V_LT_ (km·h^−1^)	14.0 ± 0.3	
G_LT_ (%)	1.6 ± 0.6	
HR_LT_ (bs·min^−1^)	179.8 ± 3.1	

Note: Values are means and SD; VO_2_max—maximal oxygen uptake; HR_max_—maximal heart rate; V_LT_—treadmill velocity at the individual anaerobic threshold; G_LT_—treadmill inclination at the individual anaerobic threshold; HR_LT_—heart rate at the individual anaerobic threshold. ^A^—values of the group in control session; ^B^—values after a series of ten WBC treatments. *—significant difference between A and B sessions (*p* ≤ 0.05).

**Table 2:** Diagram of the experimental procedure.

	Preliminary Study	Series A	10 × WBC	Series B
Exercise test	GXT test	SM test		SM test
Measurements	VO_2max_ LT HR_LT_	HR, VO_2_, RER, SBP, DBP, T_i_, T_sk_		HR, VO_2_, RER, SBP, DBP, T_i_, T_sk_
		IRT		IRT
Regions of interest in IRT		Anterior view		Anterior view

Legend: GXT test—a graded exercise test; SM test—a submaximal endurance exercise test; VO_2_max—maximal oxygen uptake; LT—anaerobic threshold; HR_LT_—heart rate at an anaerobic threshold; VO_2_—oxygen uptake; HR—heart rate; RER—respiratory exchange ratio; SBP—systolic blood pressure; DBP—diastolic blood pressure; Ti—internal temperature; Tsk—skin temperature; IRT—infrared thermography.

**Table 3:** Mean exercise workloads and physiological indicators at rest and during exercise (steady state) before (A) and after (B) a series of ten WBC interventions with corresponding statistical significance (ANOVA output) and comparisons of interest.

Indicators	A $\bar{x}$ ± SD	B $\bar{x}$ ± SD	Effect of Intervention (B) Effect of Exercise (t) Interaction (B × t); *p*; η^2^
VO_2rest_ (L·min^−1^)	0.51 ± 0.12	0.55 ± 0.09	(B); p = 0.8 (t); p = 0.001; 0.9 (B × t); p = 0.25
VO_2mean_ (L·min^−1^)	3.44 ± 0.37	3.47 ± 0.34	
HR_rest_ (bs·min^−1^)	79.2 ± 14.2	77.4 ± 14.3	(B); p = 0.29 (t); p = 0.001; 0.9 (B × t); p = 0.57
HR_mean_ (bs·min^−1^)	167 ± 7.7	169.5 ± 8.5	
VO_2max_ (%)	71.6 ± 10.9	72.0 ± 5.98	*p* > 0.05

**Table 4:** Mean physiological indicators at rest and during exercise (steady state) before (A) and after (B) a series of ten WBC interventions with corresponding statistical significance (ANOVA output) and comparisons of interest.

Indicators	A $\bar{x}$ ± SD	B $\bar{x}$ ± SD	Effect of Intervention (B) Effect of Exercise (t) Interaction (B × t) *p*; η^2^
Ti_rest_ (°C)	36.72 ± 0.31	36.49 ± 0.25	(B); p = 0.11 (t); p = 0.000; 0.6 (B × t); p = 0.26
Ti_last_ (°C)	37.96 ± 0.39	37.60 ± 0.17 *	
SBP_rest_ (mmHg)	137.5 ± 3.1	139.3 ± 3.4	(B); p = 0.97 (t); p = 0.005; 0.3 (B × t); p = 0.53
SBP_last_ (mmHg)	129.0 ± 4.2	126.8 ± 4.6	
DBP_rest_ (mmHg)	75.5 ± 2.9	73.5 ± 10.7	(B); p = 0.38 (t); p = 0.005; 0.2 (B × t); p = 0.71
DBP_last_ (mmHg)	72.2 ± 5.3	68.8 ± 8.3	
MAP_rest_ (mmHg)	94.76 ± 8.05	96.06 ± 9.34	(B); p = 0.59 (t); p = 0.004; 0.3 (B × t); p = 0.89
MAP_last_ (mmHg)	88.10 ± 8.69	89.93 ± 6.79	
$\bar{T_{\mathrm{brest}}}$ (°C)	34.62 ± 0.22	34.88 ± 0.28	(B); p = 0.12 (t); p = 0.000; 0.65 (B × t); p = 0.12
$\bar{T_{\mathrm{blast}}}$ (°C)	35.91 ± 0.28	35.92 ± 0.30	
$\bar{{\mathrm{MT}}_{\mathrm{SKrest}}}$ (°C)	31.62 ± 0.23	32.52 ± 0.45 *	(B); p = 0.003; 0.6 (t); p = 0.001; 0.8 (B × t); p = 0.34
$\bar{{\mathrm{MT}}_{\mathrm{SKlast}}}$ (°C)	33.09 ± 0.29	33.76 ± 0.68 *	
M_rest_ (W·m^−2^)	137.5 ± 3.1	145.2 ± 3.4	(B); p = 0.58 (t); p = 0.000; 0.3 (B × t); p = 0.26
M_last_ (W·m^−2^)	423.6 ± 24.2	451.9 ± 21.6	
Δg_rest_ = Ti-MTsk (°C)	4.31 ± 0.8 ^(+1.05)^	2.87 ± 1.16 *^(+0.9)^	(B); p = 0.000; 0.65 (t); p = 0.02; 0.2 (B × t); p = 0.35
Δg_last_ = Ti-MTsk (°C)	5.36 ± 0.74	3.77 ± 0.48 *^#^	
PSI	7.04 ± 0.73	6.42 ± 0.27 *	*p* < 0.05
ƒHR PSI	0.64 ± 0.07	0.68 ± 0.03	*p* > 0.05
ƒTi PSI	0.35 ± 0.07	0.34 ± 0.03	*p* > 0.05

Data are shown as means ± SDs. Ti—internal (tympanic) temperature; MTsk—mean skin temperature calculated from telethermometric measurements; Tb—mean body temperature; SDP—systolic blood pressure; DBP—diastolic blood pressure; MAP—mean arterial blood pressure; M—metabolic rate; PSI—physiological strain index; ƒHR PSI—cardiovascular fraction of the physiological strain; ƒTi PSI—thermal fraction of the physiological strain; Δg—internal-to-mean skin temperature gradient; rest—resting values; last—end values; *n* = 14 for all data except for skin and mean body temperature (*n* = 14) in A; *n* = 14 for all data in B. * *p* < 0.05; represents a significant difference within the group between session A (control; A) and session B (after WBC cryostimulation; B); ^#^ *p* < 0.05 represents a significant difference within the group (after WBC cryostimulation) between rest and last values. A two-way analysis of variance (ANOVA) with repeated measures and a one-way analysis of variance (for PSI, ƒHR PSI and ƒTi PSI) and Bonferroni’s post-hoc test were used to identify differences between A and B.

**Table 5:** Mean hormone concentrations at rest and at the end of the exercise test, before (A) and after (B) a series of ten WBC interventions, with corresponding statistical significance (ANOVA output) and comparisons of interest.

Indicators	A $\bar{x}$ ± SD	B $\bar{x}$ ± SD	Effect of Intervention (B) Effect of Exercise (t) Interaction (B × t); *p*; η^2^
CORT_rest_ (nmol·L^−1^)	702 ± 279	525 ± 191	(B); p = 0.18; (t); p = 0.003; 0.3 (B × t); p = 0.03; 0.2
CORT_last_ (nmol·L^−1^)	892 ± 319	695 ± 273	
NE_rest_ (pg·mL^−1^)	78 ± 93	77 ± 45	(B); p = 0.29 (t); p = 0.001; 0.9 (B × t); p = 0.57
NE_last_ (pg·mL^−1^)	91 ± 39	90 ± 56	
EPI_rest_ (pg·mL^−1^)	61 ± 23	32 ± 15	(B); p = 0.11 (t); p = 0.000; 0.6 (B × t); p = 0.26
EPI_last_ (pg·mL^−1^)	72 ± 22	55 ± 72	

Data are shown as means ± SDs. CORT—cortisol concentration; NE—norepinephrine concentration; EPI—epinephrine concentration; rest—resting values; last—end values; *n* = 17 for all data. A two-way analysis of variance (ANOVA) with repeated measures and Bonferroni’s post-hoc test were used to identify differences between A and B.

## Data Availability

The data presented in this study are available upon request from the corresponding author.
